# Class I HDAC Inhibition Leads to a Downregulation of FANCD2 and RAD51, and the Eradication of Glioblastoma Cells

**DOI:** 10.3390/jpm13091315

**Published:** 2023-08-27

**Authors:** Małgorzata Drzewiecka, Dominika Jaśniak, Gabriela Barszczewska-Pietraszek, Piotr Czarny, Anna Kobrzycka, Marek Wieczorek, Maciej Radek, Janusz Szemraj, Tomasz Skorski, Tomasz Śliwiński

**Affiliations:** 1 Laboratory of Medical Genetics Faculty of Biology and Environmental Protection, University of Lodz, 90-236 Lodz, Polandgabriela.barszczewska.pietraszek@edu.uni.lodz.pl (G.B.-P.);; 2 Department of Medical Biochemistry, Medical University of Lodz, 92-216 Lodz, Poland; piotr.czarny@umed.lodz.pl (P.C.);; 3 Department of Neurobiology, Faculty of Biology and Environmental Protection, University of Łodz, 90-236 Lodz, Poland; 4 Department of Neurosurgery, Surgery of Spine and Peripheral Nerves, Medical University of Lodz, University Hospital WAM-CSW, 90-236 Lodz, Poland; 5 Fels Cancer Institute for Personalized Medicine, Lewis Katz School of Medicine, Temple University, Philadelphia, PA 19140, USA

**Keywords:** glioblastoma multiforme, valproic acid, HDAC, DNA damage response, double-strand break, synthetic lethality

## Abstract

HDAC inhibitors (HDACi) hold great potential as anticancer therapies due to their ability to regulate the acetylation of both histone and non-histone proteins, which is frequently disrupted in cancer and contributes to the development and advancement of the disease. Additionally, HDACi have been shown to enhance the cytotoxic effects of DNA-damaging agents such as radiation and cisplatin. In this study, we found that histone deacetylase inhibits valproic acid (VPA), synergized with PARP1 inhibitor (PARPi), talazoparib (BMN-673), and alkylating agent, and temozolomide (TMZ) to induce DNA damage and reduce glioblastoma multiforme. At the molecular level, VPA leads to a downregulation of FANCD2 and RAD51, and the eradication of glioblastoma cells. The results of this study indicate that combining HDACi with PARPi could potentially enhance the treatment of glioblastoma, the most aggressive type of cancer that originates in the brain.

## 1. Introduction

Glioblastoma multiforme (GBM), which accounts for 54% of all gliomas, is recognized as the most aggressive primary brain tumor. With treatment, the average life expectancy for patients with glioblastoma multiforme is approximately 14–15 months Ref. [1]. Standard treatment of glioblastoma involves maximal safe surgical resection followed by radiation therapy with concurrent and adjuvant temozolomide, with or without tumor treating fields.

Synthetic lethality is a concept that has gained significant attention in cancer research and targeted therapy approaches. It refers to the phenomenon whereby the simultaneous disruption of two genes, neither of which is individually lethal, results in cell death. This concept has been particularly explored in the context of DNA repair pathways and the development of novel treatment strategies. One notable example of synthetic lethality involves the inhibition of poly (ADP-ribose) polymerase (PARP) enzymes. The PARP protein family, also known as poly (ADP-ribose) polymerases, plays a crucial role in DNA repair and genomic stability. PARP enzymes are involved in detecting and signaling DNA damage, recruiting other repair proteins to the site of damage, and facilitating the repair process. PARP inhibitors (PARPi) are a class of drugs designed to block the activity of PARP enzymes [1]. PARPi work by binding to the catalytic domain of PARP enzymes, preventing them from adding poly (ADP-ribose) chains to target proteins involved in DNA repair [2]. By inhibiting PARP activity, these drugs impair the DNA repair process, leading to the accumulation of DNA damage and, ultimately, causing cell death, particularly in cancer cells. PARPi have shown significant clinical success in the treatment of certain types of cancer, such as ovarian and breast cancer, especially in patients with specific mutations in DNA repair genes, such as BRCA1 and BRCA2. PARPi have also been investigated for their potential use in combination with other cancer therapies, such as chemotherapy and radiation therapy, in relation to their ability to enhance the effectiveness of these therapies. Ongoing research aims to further understand the mechanisms of PARPi and identify new therapeutic strategies to maximize their benefits in cancer treatment while minimizing potential side effects [2,3]. Potent anticancer effects of PARPi are being explored at various stages of preclinical and clinical trials as a combination or monotherapy in GBM tumors [4,5,6,7,8,9,10,11,12,13,14,15,16,17].

Histone deacetylases (HDACs) are a family of enzymes that have an essential role in controlling gene expression and modifying chromatin [18]. The main function of HDACs is to eliminate acetyl groups from histone proteins, leading to the condensation of chromatin structures and the suppression of gene transcription [19]. However, aberrant HDAC activity has been implicated in various diseases, including cancer and neurodegenerative disorders. Inhibition of HDACs has emerged as a promising therapeutic strategy for the restoration of normal gene expression patterns and provides a potential treatment for these diseases [20,21]. Histone deacetylase (HDAC) activity, by the use of various small molecule chemical HDACi in combination with existing anticancer treatments, has been emerging as a promising anticancer strategy. A few of the HDACi in clinical trials for treating GBM and other brain tumors exhibit potent anticancer activity in combination with radio- and/or chemotherapy [22,23]. The effectiveness of histone deacetylase inhibitors (HDACi) in combination chemotherapy may be attributed, in part, to their ability to induce DNA double-strand breaks (DSBs). HDACi can cause changes in chromatin structure, leading to the direct activation of the DNA damage response [11,12]. This, in turn, affects the acetylation status of proteins involved in various DNA repair mechanisms and can impact the genomic stability of cancer cells [13]. Several studies have explored the effects of HDACi on genomic integrity and their interactions with poly (ADP ribose) polymerase (PARP) inhibitors (PARPi) in solid tumors [14,15,16,17,18]. PARP inhibitors have been developed as a targeted therapy for cancer treatment because they exploit the concept of synthetic lethality. In cells with deficient homologous recombination repair, such as those with BRCA mutations, inhibition of PARP leads to the accumulation of DNA single-strand breaks that are converted into more toxic DNA double-strand breaks during replication. Cancer cells with this repair deficiency are then selectively killed, due to their inability to repair the induced DNA damage, while normal cells with functional homologous recombination repair remain relatively unaffected. Given the impact of HDACi on DNA repair and genomic stability, there is a possibility that HDACi could influence the process of PARylation, either directly or indirectly, and this may contribute to their effectiveness in combination with PARPi in cancer treatment. However, more research is needed to fully understand the complex interplay between HDACi PARP inhibitors in the context of DNA repair and cancer therapy.

Based on extensive clinical experience in treating epilepsy, valproic acid (VPA) has been found to be a safe drug with excellent bioavailability [24]. Recent clinical trials conducted for different types of cancers have demonstrated that the serum concentration of VPA, achieved during epilepsy treatment with a daily dose, acts as a potent inhibitor of HDACs that are crucial for histone acetylation. The inhibitory effect of VPA on HDACs leads to an increase in histone acetylation levels, resulting in chromatin relaxation and the activation of silenced genes [24,25]. By modulating gene expression, VPA has shown potential in diverse therapeutic applications. Apart from its antiepileptic properties, VPA has been investigated for its anticancer effects. It has demonstrated the ability to induce differentiation, inhibit cell proliferation, and promote apoptosis in various cancer cell types [18]. Furthermore, VPA has been explored for its neuroprotective properties in neurodegenerative disorders such as Alzheimer’s disease and Parkinson’s disease. Studies have shown that VPA treatment can enhance neuronal survival, reduce neuroinflammation, and improve cognitive function in animal models of these diseases [19].

In our previous studies, we demonstrated that HDAC inhibitors effectively reduce the viability of melanoma cells and may render this type of cancer more susceptible to the cytotoxic effects of DNA-damaging agents, such as dacarbazine [26]. However, whether HDAC inhibitors could sensitize LIG4-deficient glioblastoma to PARPi and TMZ remains to be investigated. In this article, to explore this aspect, we use a class I HDAC inhibitor, VPA, in combination with BMN-673, a PARP1 inhibitor, and the alkylating agent TMZ. At the molecular level, we assess the impact of these HDACi treatments on the expression of FANCD2 and RAD51, molecules highly involved in HR repair [27,28,29].

## 2. Materials and Methods

### 2.1. Cell Lines and Culture Conditions

Glioblastoma specimens were obtained from patients of the Department of Neurosurgery, Surgery of Spine and Peripheral Nerves at the University Hospital WAM-CSW Łódź. The cell cultures derived from these glioblastoma specimens were established in the Laboratory of Molecular Genetics at the University of Lodz. These cell cultures were given the names GMB113 and GBM114 for identification purposes. To initiate the cell cultures, the glioblastoma tissue fragments were washed multiple times and then minced using a scalpel. The resulting cells were filtered through a cell strainer with a pore size of 70 μM. The glioblastoma cells were then cultured in DMEM medium (Lonza, Basel, Switzerland) supplemented with 10% FBS (Lonza), 100 IU/mL penicillin, 100 μg/mL streptomycin (Lonza), and 50 μg/mL gentamicin (Lonza). The cultures were maintained in a humidified atmosphere with 5% CO2 at a temperature of 37 °C. In addition to the glioblastoma cell cultures, normal human astrocytes (NHA) obtained from Lonza were also used in the study. The NHA cells were grown in ABM Basal Medium supplemented with FBS 15 mL/L, L-Glutamine 5 mL/L, GA-1000 500 µL/L; Ascorbic Acid 500 µL/L; HEGF 500 µL/L; and Insulin 1.25 mL/L.

### 2.2. Drug Treatment

Normal astrocytes and glioblastoma cells were plated in a 6-well plate at a density of 2 × 10^5^ viable cells per well. Cells were cultured with 50 nM BMN673 (Selleckchem, United States), 6.25 μM TMZ (Sigma-Aldrich, Burlington, MA, USA,), and BMN673 + temozolomide for 72 h of incubation. Valproic acid sodium salt (VPA) from SigmaAldrich was dissolved in dH_2_O to create a solution with a concentration of 100 mmol/L. This solution was then stored at −80 °C after undergoing sterile filtration. In the experiment, cells were pretreated with VPA at a concentration of 1 mmol/L for a duration of 168 h. The VPA-containing medium was refreshed every 48 h, and any remaining VPA was removed before subjecting the cells to further treatment. The treatment scheme and concentrations of the compounds used were applied in previous experiments [21,26]. The achieved micromolar doses in our experiments are significantly lower than those currently used in clinical practice. This can offer hope that patients’ exposure to pretreatment with valproic acid will sensitize them to the effects of chemotherapeutics. However, this hypothesis requires further investigation on a larger number of cell lines and in vivo studies.

### 2.3. Proliferation Determination

Cells proliferation was tested by a clonogenic assay. Cells were seeded (3 × 10^4^ cells/well) into a 12-well plate and pretreated with VPA (10 mM) (for 168 h); then, they were treated with BMN-673 (50 nM), either used alone or in combination with TMZ (6.25 μM) (for 72 h). After treatment, cells were resuspended in 700 µL of soft agar (DMEM, 0.4% *w*/*v*) and reseeded over 700 µL of solidified agar underlay (DMEM, 0.5% agar) into a 6-well plate (2 × 10^3^ cells/well). After solidifying, the cell layer was covered with medium (changed weekly). After two weeks, cells were washed gently with PBS (Phosphate-Buffered Saline). After the washing step, the cells were fixed and stained using a DNA intercalating dye, specifically crystal violet (0.5% *w*/*v*), for a minimum of 30 min. To remove the excess stain, a technique involving the gentle dunking of the plates in beakers filled with water was employed. This process continued until all excess stain was eliminated, leaving only bright-purple colonies on the plates. Cells were counted under the microscope, and clonogenic efficiency was expressed as percent of untreated control (no. of colonies after treatment vs. no. of colonies in control sample × 100%).

### 2.4. Cell Viability

Glioblastoma cells were seeded at a density of 7 × 10^3^ viable cells per well in a 12 well-plate. Then, the cells were pretreated with 1 mM valproic acid for a duration of 168 h, with a refreshment of VPA-containing medium every 48 h. After a 168-h pretreatment period, the cells were cultured with 50 nM BMN-673 (MedChemExpress; Cat#HY-16106), 6.25 μM TMZ (Sigma-Aldrich, Burlington, MA, USA), either used alone or in combination. To determine cell viability after treatments with VPA, TMZ, and BMN673, a trypan blue exclusion assay was performed. Cells were counted (within 3 to 5 min of mixing with 0.4% trypan blue) by light microscopy, using a Neubauer hemocytometer. The experiments were carried out three times, in triplicate.

### 2.5. RNA Isolation and Real-Time Polymerase Chain Reaction

In this study, RNA was isolated from cultured GBM113 and GBM114 cell pellets containing approximately 2.5 × 106 cells. The extraction was performed using a total RNA isolation kit (A&A Biotechnology; Cat#031-100). Subsequently, the RNA samples were converted into complementary DNA (cDNA), using SuperScript II Reverse Transcriptase from Invitrogen Life Technologies. For quantitative reverse transcription PCR (qRT-PCR), TaqMan Real-Time PCR Master Mix from Life Technologies was utilized. The qPCR reactions were conducted on an Agilent Technologies Stratagene M × 3000 P system with MxPro software. The expression levels of seven genes, which were involved in DNA double-strand break repair pathways, were examined using TaqMan probes from Life Technologies. To ensure accurate normalization of the expression data, 18 S RNA was employed as the reference gene. TaqMan probes from Life Technologies were also utilized for this purpose. The qPCR cycling parameters consisted of an initial step of 95 °C for 10 min, followed by 30 cycles of denaturation at 95 °C for 15 s and annealing/extension at 60 °C for 60 s.

### 2.6. Comet Assay

The Comet assay was conducted following the methodology outlined in a previous study [30]. Cells were cultured for a period of 72 h in the presence of either drugs or a vehicle. Prior to the 72-h culture, the cells were pretreated with VPA (Valproic acid) for a duration of 168 h. Fifty comet images were randomly selected for each treatment variant and the percentage of DNA in the tail (% tail DNA) was measured. The mean value for this parameter was taken as an index of DSBs in the given sample.

### 2.7. Apoptosis and Necrosis

To assess apoptosis and necrosis in the cell population, FITC Annexin V staining was performed, along with a vital dye such as propidium iodide (PI) or 7-Amino-Actinomycin (7-AAD). This staining method allows for the identification of early apoptotic cells (PI negative, FITC Annexin V positive) before the loss of membrane integrity that occurs in the later stages of apoptotic or necrotic cell death. In this study, a FITC Annexin V Apoptosis Detection Kit from BD Biosciences (Catalog #556547) was used to quantitatively determine the percentage of cells actively undergoing apoptosis, following the manufacturer’s instructions. The experimental procedure involved plating the cells at a density of 1 × 10^5^ cells per well in a 24-well plate. The cells were then pretreated with 1 mM valproic acid (VPA) for 168 h, with a refreshment of VPA-containing medium every 48 h. Subsequently, the VPA-containing medium was removed before further treatment. After the 168-h pretreatment period, the cells were cultured with 50 nM BMN-673 from MedChemExpress (Catalog #HY-16106) and 2 m TMZ from Sigma-Aldrich, Burlington, MA, USA. These treatments were administered individually or in combination. Following a 24-h incubation with VPA, BMN-673, and TMZ, the cells were washed twice with cold PBS and resuspended in 1xBinding Buffer at a concentration of 1 × 10^5^ cells/mL. Then, 100 μL of the solution (1 × 10^4^ cells) was transferred to a 5 mL culture tube. FITC Annexin V (5 μL) and PI (5 μL) were added to the tube. The cells were gently vortexed and incubated in the dark at room temperature (25 °C) for 15 min. Finally, 400 μL of 1xBinding Buffer was added to each tube, and the samples were analyzed by flow cytometry within 1 h.

### 2.8. Histone H2AX

The GBM113 and GBM114 cell line was cultured at a density of 5 × 10^3^ cells per well. To detect the levels of phosphorylated Histone H2AX, the H2AX Phosphorylation Assay Kit (Flow cytometry) from Millipore (Catalog #17-344) was utilized. The assay was performed on cultured cells that were treated with agents capable of inducing DNA damage or apoptosis. Prior to treatment, the cells were pretreated with 1 mM valproic acid (VPA) for 168 h, with a refreshment of VPA-containing medium every 48 h. Subsequently, the VPA-containing medium was removed before further treatment. After the 168-h pretreatment period, the cells were cultured with 50 nM BMN-673 and 6.25 μM TMZ, which promoted H2AX phosphorylation. These mentioned agents were used either individually or in combination. Following treatment, the cells were fixed and permeabilized to facilitate staining and detection. The presence of Histone H2AX phosphorylated at serine 139 was detected using a FITC-conjugated anti-phospho-Histone H2AX antibody. Flow cytometry was employed to quantify the number of cells exhibiting positive staining for phosphorylated Histone H2AX.

### 2.9. Cell Cycle

For the analysis of cell cycle distribution, GBM113 and GBM114 cells, either untreated or treated, were subjected to propidium iodide (PI). After a 48-h treatment period, the cells were washed with cold 1× PBS and fixed in 70% ethanol on ice for at least 1 h. Following centrifugation, the cell pellet was washed with cold 1× PBS and stained with a solution containing 50 μg/mL PI and RNase, incubated for 15 min at 37 °C, and subsequently analyzed using FACSCalibur (BD Biosciences, Franklin Lakes, NJ, USA).

### 2.10. Statistical Analysis

All graphs show the mean ± standard deviation (SD), and statistically significant differences were analyzed using Student’s *t*-test with GraphPad Prism 7 (GraphPad Software, Inc., San Diego, CA, USA).

## 3. Results

### 3.1. Expression of Genes Involved in DSB Repair in Normal Human Astrocytes and Glioblastoma Cells

**Figure 1:**
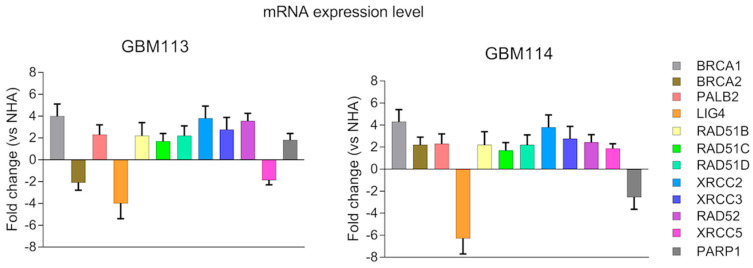
Expression profiles of 12 specified genes involved in HR (homologous recombination), D-NHEJ (DNA-dependent non-homologous end joining), and B-NHEJ (backup non-homologous end joining) repair systems were compared between glioblastoma cells and normal human astrocytes. The expression levels of these genes in primary human glioblastoma cell lines (GBM113 and GBM114) were normalized to the expression of the reference gene, 18 S rRNA. The data are presented as fold changes relative to normal human astrocytes (NHA). The results represent the mean value ± standard deviation (SD) obtained from three independent experiments, each performed in triplicate.

To implement a personalized synthetic lethality approach, we conducted an analysis of gene expression profiles in two patient-derived glioblastoma primary cell lines and compared them with the profile of normal human astrocytes (NHA). Our focus was on 12 genes involved in DNA double-strand break (DSB) repair pathways. These genes included BRCA1, BRCA2, PALB2, RAD51B, RAD51C, RAD51D, XRCC2, XRCC3, RAD52 (part of HR—homologous recombination pathway), LIG4, XRCC5 (part of D-NHEJ DNA-PK dependent non-homologous end-joining pathway), and PARP1 (part of B-NHEJ backup non-homologous end-joining pathway). Significant changes in the mRNA expression profile of LIG4 was observed between the glioblastoma cell lines and NHA, as shown in Figure 1.

### 3.2. Response of Patient-Derived Glioblastoma Cells and Normal Astrocytes to VPA Inhibitor Used Alone or in Combination with Alkylating Agent and PARP1 Inhibitor

**Figure 2:**
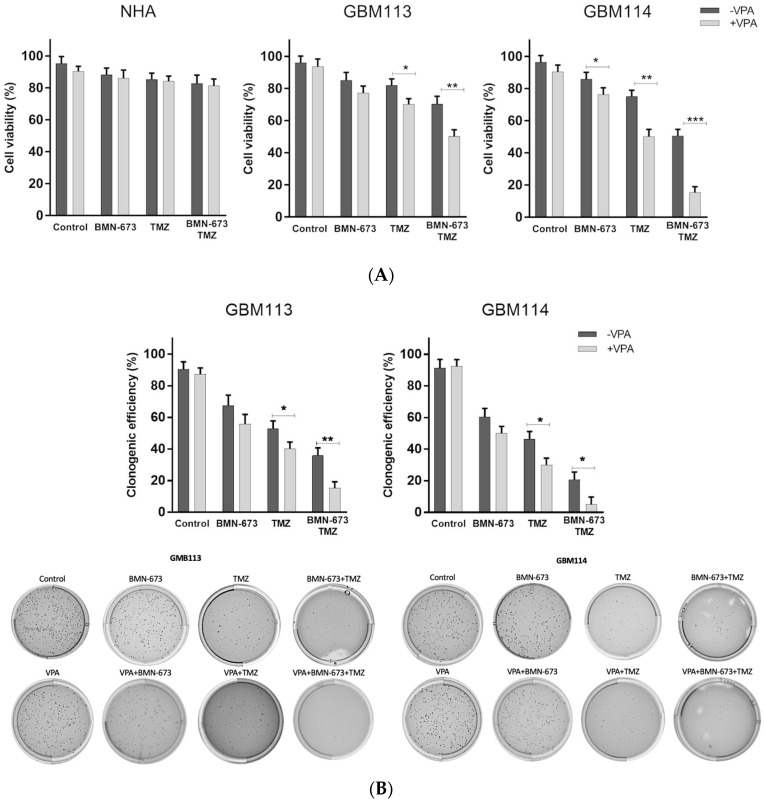
The effect of VPA, BMN-673, and TMZ treatment on cell viability (**A**) and proliferation (**B**) after 240-h incubation (168-h pretreatment with VPA and 72-h incubation with BMN-673 and TMZ). At least three independent experiments were performed, and the results are shown as the mean ± standard deviation (SD). * *p* ≤ 0.05 and ** *p* < 0.001, *** *p*-value ≤ 0.001 when compared to control group.

Next, we investigated whether the pretreatment with HDAC inhibitor alters the cytotoxicity induced by TMZ and BMN-673. GBM113 and GBM114 cell lines were treated with appropriate IC50 concentrations of each drug, either alone or in various combinations, for 240 h: 168 h of pretreatment with VPA (10 mM), followed by 72 h of 6.25 μM TMZ and 50 nM BMN-673 [30]. When compared to individual agents, the combination of VPA, BMN 673, and TMZ exerted significantly stronger anti-GBM113 and -GBM114 glioblastoma effects, with only minimal toxicity to normal astrocytes. Cell viability was determined using trypan blue staining. Clonogenic assay was used to examine the influence of drugs on the colony-forming ability of cancer cells. VPA, in combination with BMN-673 or TMZ, had an influence on clonogenic efficacy, while the triple combination of VPA + BMN673 + TMZ was able to almost completely abolish the clonogenic growth of LIG4-deficient glioma cells (Figure 2).

### 3.3. Valproic Acid Downregulates RAD51 and FANCD2

**Figure 3:**
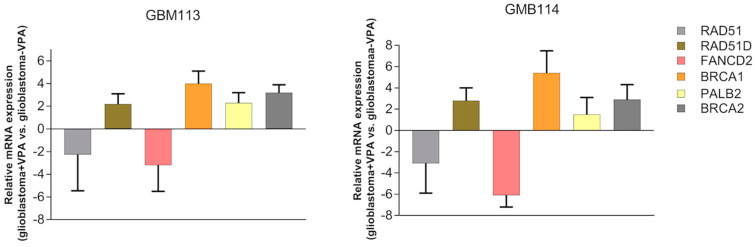
VPA downregulates RAD51 and FANCD2 in glioblastoma cells. GMB113 and GBM114 cells were treated with VPA (10 mM) for 168 h. Real-time PCR results showing the down- and upregulation of the listed genes.

To examine how inhibiting class I HDACs with VPA enhances sensitivity to temozolomide and BMN-673, a real-time PCR array was conducted in GMB113 and GBM114 glioblastoma cells. The aim was to monitor changes in the mRNA expression of six genes (chosen based on previous publications [21,26]) involved in the double-strand break repair pathway (RAD51, RAD51D, FANCD2, BRCA1, BRCA2, PALB2). In Figure 3, the results of the PCR array showed alterations in the mRNA expression profile of RAD51 and FANCD2. The findings revealed that RAD51 and FANCD2 protein levels decreased following inhibition of HDACs.

### 3.4. Inhibition of Class I HDACs Increases the Number of DSBs in Glioblastoma Cells

**Figure 4:**
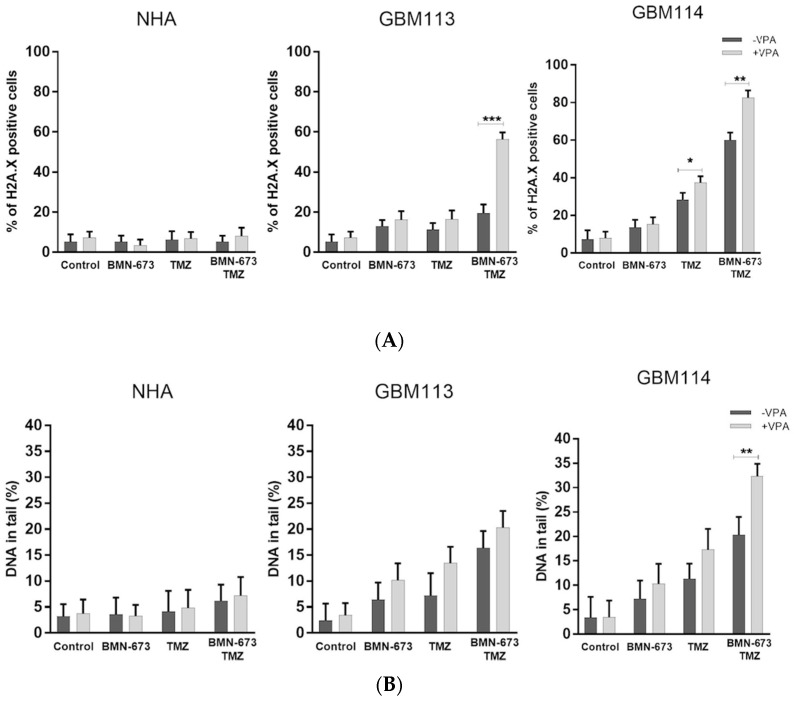
Accumulation of DSB in VPA + BMN673 + TMZ-treated GMB113 and GBM114 glioblastoma cells. (**A**) DSBs were detected by γH2A.X immunofluorescence. Bars show mean percentage of γH2A.X—positive cells ± SD from 3 independent experiments. (**B**) DSBs were detected by neutral comet assay. Bars show mean percentage of DNA in tail ± SD from 50 randomly selected cells in 3 independent experiments. * *p* < 0.05 and ** *p* < 0.001, *** *p*-value ≤ 0.001 when compared to control group.

GBM113 and GB114 cells showed elevated levels of γ-H2AX after treatment with the tested compounds (Figure 4A). Furthermore, when GMB113 cells were treated with a combination of VPA, TMZ, and BMN-673, there was approximately a twofold increase in phosphorylation levels of H2AX compared to treatment with either drug alone. In NHA cells, the level of γH2A.X-positive cells stayed at relatively low level, regardless of the treatment used. The neutral comet assay was utilized to identify DNA double-strand breaks (DSBs) following the administration of VPA, BMN673, and/or TMZ. The combination treatment involving VPA, BMN673, and TMZ resulted in a substantial and notable elevation in DSBs in both glioblastoma cell lines.

### 3.5. Effects of Valproic Acid on the Cell Cycle and Apoptosis

**Figure 5:**
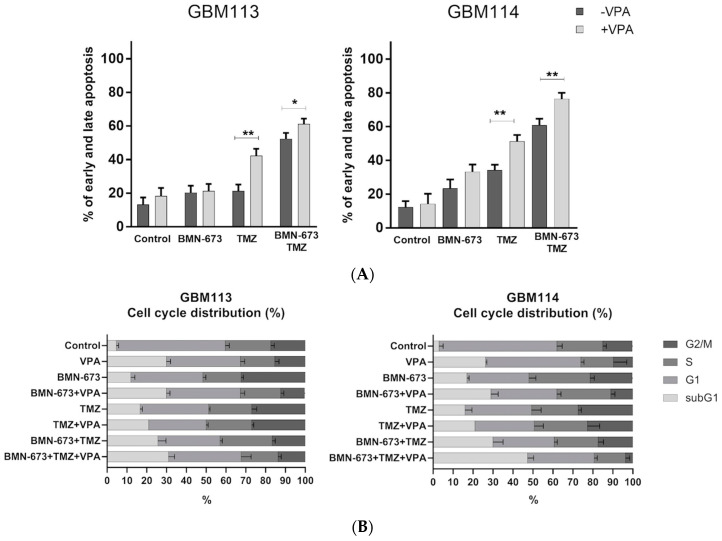
Effects of valproic acid on the cell cycle and apoptosis (**A**). The percentage of GBM113 and GBM114 glioblastoma cells in early and late apoptosis (**B**). Cell cycle analysis was evaluated by FACS analysis after 48 h of treatments, following staining with PI. The bars represent the mean of the percentage of cells in each phase of cell cycle (subG1, G1, S and G2) plus S.D. of three experiments. At least three independent experiments were performed, and the results are shown as the mean ± standard deviation (SD). * *p* ≤ 0.05, ** *p* < 0.001, compared with the control group.

In order to analyze the potential anti-glioblastoma effect of the HDAC inhibitor, either used alone or in combination with a PARP1 inhibitor and an alkylating agent, a double-staining method utilizing propidium iodide (PI) and Annexin V was employed (Figure 5A) This staining technique allows for differentiation between live and dead cells, as well as early apoptotic vs. late apoptotic/necrotic cells. The combination of VPA + BMN673 + TMZ exhibited a significantly stronger anti-glioma effect on GBM113 and GBM114 cells. Flow cytometry analysis indicated that cell death primarily occurred through the process of apoptosis. Such an effect was accompanied by an increase in subG1 events in both cell lines, providing confirmation of a more potent pro-apoptotic effect (Figure 5B).

## 4. Discussion

The involvement of HDICs in cancer has raised hope that these enzymes may represent valuable targets in drug discovery programs. Recent clinical trials demonstrated that, at least for hematological cancers, small molecules that inhibit HDACs can be effective pharmacological agents when administered, either alone or in combination with other drugs. HDACi exhibit a number of anti-proliferative effects, such as cell cycle arrest, differentiation, angiogenesis inhibition, and apoptosis. A significant number of HDACi, such as suberoylanilide hydroxamic acid (SAHA, also known as vorinostat or Zolinza), entinostat (MS-275), romidepsin (Istodax), and belinostat (PXD-101) are at various stages of drug development. The use of HDAC inhibitors (HDACi) has proven to be an effective strategy in anticancer treatment, both as single agents and in combination therapies targeting various types of cancer, including pancreatic, solid tumors, and hematologic malignancies. HDAC inhibitors have demonstrated their ability to interfere with DNA repair processes [22], leading to their incorporation into combination therapies with DNA-damaging agents such as cisplatin, doxorubicin, and ionizing radiation. Furthermore, HDAC inhibitors have been shown to enhance the sensitivity of cancers, such as triple-negative breast cancer and prostate cancer, to PARP inhibitors, suggesting their potential in combination therapies [31].

PARP inhibition has been shown to be efficient in eradicating GBM cells in vitro and in vivo, either alone or in combination with chemo-radiation [32]. Moreover, several clinical trials evaluating a number of PARP inhibitors are ongoing, with the hope for significant improvement of GBM patients’ survival. Histone deacetylases (HDACs) have recently become recognized as a promising target for cancer therapy, including for the treatment of GBM. HDACs, together with histone acetylases (HATs), are responsible for chromatin structure remodeling, thereby regulating the expression levels of numerous genes essential for cancer cell survival. PARP inhibitors have demonstrated their effectiveness in eliminating GBM (glioblastoma multiforme) cells both in laboratory settings (in vitro) and in live organisms (in vivo), either when used alone or in combination with chemo-radiation. There have been successful studies conducted by Dungey et al. (2009) supporting this claim. Encouraged by these results, multiple clinical trials are currently underway, investigating various PARP inhibitors with the hope of significantly improving the survival rates of GBM patients (source: clinicaltrials.gov). Histone deacetylases (HDACs) have emerged as a promising target for cancer therapy, including the treatment of GBM. HDACs, along with histone acetylases (HATs), play a crucial role in remodeling the structure of chromatin, which ultimately regulates the expression of numerous genes essential for cancer cell survival. SAHA (Vorinostat) is the first FDA-approved HDAC inhibitor, and it has been successfully employed in treating cutaneous T-cell lymphoma. This success has encouraged the development of other small molecule HDAC inhibitors for potential clinical use, as indicated in the study by Jain et al. [33]. However, it is worth noting that HDAC inhibitors, although promising in various cancer types, are not as effective as monotherapy against solid tumors such as GBM. This limitation is partly attributed to the poor pharmacokinetic properties of these inhibitors, as observed in studies conducted by Park et al. [34]. Thus, further research and development are needed to optimize the potential of HDAC inhibitors as a standalone treatment for solid tumors such as GBM. Hence, it is of great clinical significance to identify potential sensitizers that can enhance the effectiveness of treatment. Several HDAC inhibitors have been proposed as candidates, as they have the capability to disrupt the functionality of the HR (homologous recombination) pathway, leading to a phenotype resembling HR deficiency. This similarity increases the sensitivity of PARP inhibitors, making them more effective in their action. Studies conducted by Ha et al. [35], Mendes-Pereira et al. [36], and Min et al. [37] have provided evidence supporting this notion. These findings highlight the potential of using HDAC inhibitors as a strategy to improve PARP inhibitor response and, ultimately, enhance the treatment outcomes for diseases such as GBM.

LIG4 is an essential component of the DNA non-homologous end-joining (D-NHEJ) pathway, and its decreased levels can lead to impaired functioning of this repair system. Previous studies have indicated that downregulation of LIG4, a gene-encoding LIG4, has been observed in high-risk neuroblastomas derived from patients. This downregulation has been associated with more advanced stages of the disease and lower chances of survival [22]. Additionally, research on glioblastoma cohorts has also shown reduced expression of LIG4, but the specific mechanisms responsible for this reduced expression have not been identified. However, a recent study suggested that inefficient JAK2-STAT5 and/or PI3K-AKT pathways might be involved in regulating the expression of LIG4 [23]. These findings highlight the potential significance of LIG4 in cancer development and may have implications for therapeutic approaches targeting the D-NHEJ pathway in certain cancers.

We propose that a deficiency in the DNA non-homologous end-joining (D-NHEJ) pathway due to the downregulation of LIG4 could lead to a synthetic lethal interaction with the deficiency in the backup non-homologous end-joining (B-NHEJ) pathway induced by PARP inhibitors (PARPi) in glioblastoma cells that experience DNA damage from temozolomide (TMZ) treatment. Supporting this hypothesis, we have demonstrated that melanoma cells lacking LIG4 were highly susceptible to a combination of the alkylating agent dacarbazine and PARPi [17]. This suggests that glioblastoma cells with reduced LIG4 expression may display increased sensitivity to PARPi when exposed to TMZ-induced DNA damage, potentially leading to a selective targeting of these cancer cells. The synthetic lethal interaction between D-NHEJ and B-NHEJ deficiencies could be exploited as a therapeutic strategy to enhance the efficacy of PARPi-based treatments in certain types of cancers. However, further research is required to validate and understand the mechanisms underlying this potential synergistic effect. In our study, we discovered that the combination of PARP inhibitors (PARPi) and HDAC inhibitors (HDACi) holds promise as a potential therapeutic approach for glioblastoma. VPA, which selectively inhibits class I HDACs, demonstrated increased cytotoxicity when combined with BMN-673, a PARP1 inhibitor and alkylating agent. This enhanced cytotoxic effect was correlated with the induction of more pronounced DNA damage in cells treated with the HDACi/PARP1 combination, attributed to the downregulation of FANCD2 and RAD51 by VPA. In the first part of our study, we investigated the response of patient-derived glioblastoma cells and normal astrocytes to the VPA inhibitor, both as a single agent and in combination with an alkylating agent and PARP1 inhibitor. Our findings demonstrated that, while VPA sensitized the treated cells, the triple combination exhibited the most significant efficacy.

Apoptosis is a crucial process characterized by biochemical and morphological changes that ultimately lead to cell death. The TP53 DNA damage checkpoint tumor suppressor genes, regardless of the specific tumor, are among the most frequently mutated genes. Apoptosis initiation relies on the delicate balance between two apoptosis regulators, Bcl-2 and Bax, wherein maintaining the appropriate expression levels of these proteins is essential for cell survival. Higher expression of Bcl-2 inhibits apoptosis [38,39]. Common anticancer therapies, including chemotherapy and radiotherapy, induce apoptosis in various cancer cells. Literature suggests that DNA damage or damage to other critical molecules is often the initial trigger in the apoptotic process. It was observed that each of the three compounds tested in this study induces apoptosis and increases the levels of phosphorylated H2A.X, thereby influencing the expression of genes involved in HR repair in glioblastoma cell lines. Other studies have shown that the tested inhibitors, PARP1 and HDAC, induced apoptosis in DMBC11, H6, and H7 cell lines by causing DNA damage [26,30,38]. Similarly, Romeo et al. reported that VPA and TSA sensitized pancreatic cancer cells to an AZD2461 PARP inhibitor. They proposed that the mechanism of action is associated with the downregulation of CHK1 and RAD51 [39]. The heightened cytotoxicity achieved by the combination of HDACi and PARPi and alkylating agent may be attributed to the impairment of the HR DNA repair pathway, a mechanism often associated with resistance to PARP inhibitor treatment [21].

## 5. Conclusions and Limitations

In summary, our findings provide support for the idea that combining therapies against glioblastoma could be an effective approach to improve outcomes. While more research is necessary to validate the efficacy of this combination strategy, the results suggest that it holds promise as a multimodal therapy for GBM. It is important to note that, although pre-clinical studies have shown encouraging results, the effectiveness of HDAC inhibitors in clinical practice will require confirmation through larger prospective trials. Because, in our study, we examined only two cell lines that were responsive to treatment, additional research is required to investigate the epigenetic mechanisms driving the pathogenesis of glioblastoma multiforme, especially its resistance to treatment, and to identify molecular subtypes, particularly those resistant to therapy, with a focus on RAD18 gene expression levels. This area of investigation holds great promise for future research [40].

## Data Availability

The data presented in this study are available upon request.
